# Effects of Nanocylinders on the Whispering Gallery Modes in a Microcylinder

**DOI:** 10.3390/s16040512

**Published:** 2016-04-09

**Authors:** Jinwoo Han

**Affiliations:** Department of Physics, Daegu University, Gyungsan 712-714, Korea; hanwoo@daegu.a.c.kr; Tel.: +82-53-850-6437

**Keywords:** microcylinder resonator, whispering gallery mode, nanocylinder, optical modes

## Abstract

Optical biosensors have been studied extensively for the detection and characterization of biological entities, such as viruses, bacteria, and biomolecules. A two-dimensional (2D) microcylinder resonator ($Q∼2\times 10^{5}$) was designed, and the effects of a nanocylinder on the whispering gallery modes (WGMs) were examined numerically. For this purpose, the finite element method with COMSOL multiphysics software was employed. The perturbation of the WGM resonances can be characterized by the shift and splitting of the resonance peaks, which varies according to the position, size, and refractive index of an embedded nanocylinder. The positional dependence shows a large splitting in the region of strong electric fields, and the size dependence shows a broad peak of the splitting at $R_{c}=110\;\text{nm}$. These results are attributed to the changing degree of overlap of the WGMs with the nanocylinder. The refractive index dependences of splitting show linear behavior for a nanocylinder less than 50 nm in size, and the nonlinear behavior increases with increasing size of the nanocylinder. The optical resonator system is shown to be suitable for detecting impurity particles, which are smaller than the sizes of the node and antinode regions.

## 1. Introduction

Optical biosensors have been studied extensively for the detection and characterization of biological nanoparticles, such as viruses, bacteria, and biomolecules [1,2,3,4,5,6]. In a microdisk resonator, light circulates along the curved path near the boundary in the form of the whispering gallery modes. If nanopores or nanoparticles are present along the light path, light interacts with the nanosize objects. As a result of this interaction, the whispering gallery mode (WGM) splits into two counter-circulating WGM modes, eventually resulting in the formation of two standing wave modes. It has been shown that nanosize objects can be characterized by the shift in the resonance wavelength and the splitting between the resonance peaks.

For example, Arnold *et al.* studied the shift of WGM peaks caused by protein on the spherical WGM resonator [1]. Resonance shifts of WGMs by nanoparticles near the spherical WGM resonator also studied theoretically [7,8]. Kim *et al.* used silica microtoroids to study the peak splitting caused by polystyrene nanoparticles, 50 nm in radius [9]. Zhu *et al.* demonstrated the detection and sizing of single influenza A virions, polystyrene, and gold nanoparticles using peak splitting in an ultra-high-Q resonator [10]. Yi *et al.* investigated theoretically the mode splitting in a high-Q WGM microresonator coupled to multiple subwavelength Rayleigh scatters [11]. Hiremath examined the perturbations of WGMs by embedded particles in cylindrical microcavities [12]. In addition, many numerical studies have been carried out previously [13,14,15,16].

In the present study, we designed a two-dimensional (2D) microcylinder resonator $\left(Q\sim 2\times 10^{5}\right)$ coupled to a waveguide. The main objective of this study was to investigate the effects of a nanocylinder, embedded below the boundary surface of a microcylinder on the WGMs. The main focus was on the perturbation of the WGM resonances by varying the position, size, and refractive index of the nanocylinder.

## 2. Materials and Simulation Methods

The finite element method (FEM) was used to simulate the WGMs in the microcylinder resonators. COMSOL Multiphysics with a RF module (Ver. 4.1, COMSOL Inc., Burlington, MA, USA) was used for numerical analysis of a two-dimensional (2D) model system and post-processing.

**Figure 1:**
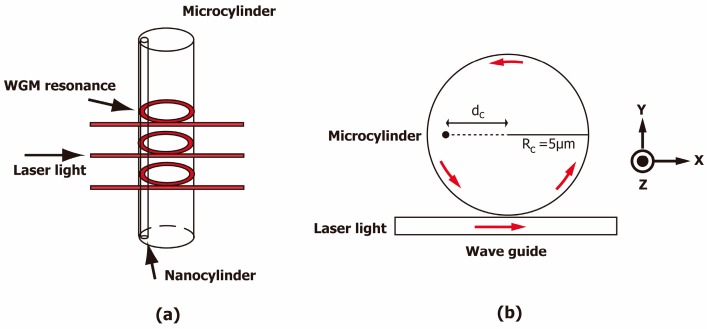
(Color online) Schematic diagram of the microcylinder resonator and bus waveguide (**a**) in 3-D and (**b**) in 2-D. A nanocylinder is located at a distance $d_{c}$ from the center.

A microcylinder resonator coupled to a bus waveguide was designed, as shown in Figure 1. The microcylinder resonator and the bus waveguide are made from Si_3_N_4_. The surrounding material for the microcylinder and the bus waveguide is assumed to be air. The refractive indices of the microcylinder (Si_3_N_4_) and air are n_mc_ = 2.01 and n_air_ = 1, respectively.

The diameter of the microcylinder resonator is 10μm, and the width of the waveguide is 2 μm. The gap between the resonator and the waveguide is 500 nm. The simulation domain is set to a 14 μm × 16 μm rectangular domain. The input port is excited optically with a plane wave that is directed in the x-direction and polarized in the z-direction. Optical excitation is provided through the input port at the left end of the wave guide, as shown in Figure 1.

The wavelength of the incident optical signal varies from 800 to 802 nm. The resonance spectra are obtained by calculating the electromagnetic energy density of the microdisk as a function of the wavelength. It should be noted that the wavelength, *λ*, is the value in free space, unless otherwise specified. A nanocylinder is embedded at a position of $d_{c}$ from the center, as shown in Figure 1.

## 3. Results and Discussion

**Figure 2:**
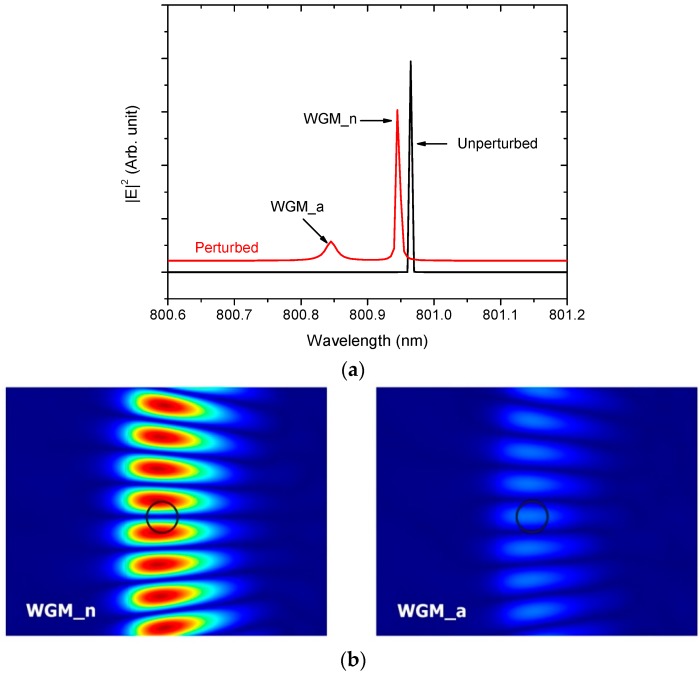
(Color online) (**a**) Representative spectra of the whispering gallery mode (WGM) resonances (unperturbed and perturbed by a nanocylinder of 50 nm in radius). The spectra are displaced vertically for clarity; (**b**) Electric field distributions at the position of the nanocylinder (100 nm in radius) for the two split WGMs.

This study examined the effects of a nanocylinder embedded inside a microcylinder on the WGMs. Figure 2a shows the representative spectra of the WGM resonances, unperturbed and perturbed by a nanocylinder, 50 nm in radius. Perturbation of the WGM by the nanocylinder causes a shift in the resonance wavelength and splitting of each unperturbed resonance. In this case, one peak is blue-shifted considerably (broad), and the other is blue-shifted slightly (narrow), as shown in Figure 2a. The two peaks are denoted as WGM_a and WGM_n, respectively.

According to the distributions of the electric field for the two split WGMs, the WGM_a corresponds to the anti-node position of the nanocylinder, whereas the WGM_n corresponds to the node position of the nanocylinder. The resonance wavelengths of these peaks are denoted as $\lambda_{a}$ and $\lambda_{n}$, respectively. The resonance wavelength of the unperturbed WGM is $\lambda_{o}=800.966\;\mathrm{nm}$. The WGM_a is perturbed more intensely by the nanocylinder than the WGM_n because the electric fields of the WGM_a have a maximum overlap with the nanocylinder, whereas those of the WGM_n have a minimum overlap. Consequently, the two peaks are blue-shifted by different amounts. The peak splitting, denoted as $\delta \lambda =\lambda_{n}-\lambda_{a}$, can be used to measure the perturbation of the WGMs by the nanocylinder.

### 3.1. Positional Dependence

**Figure 3:**
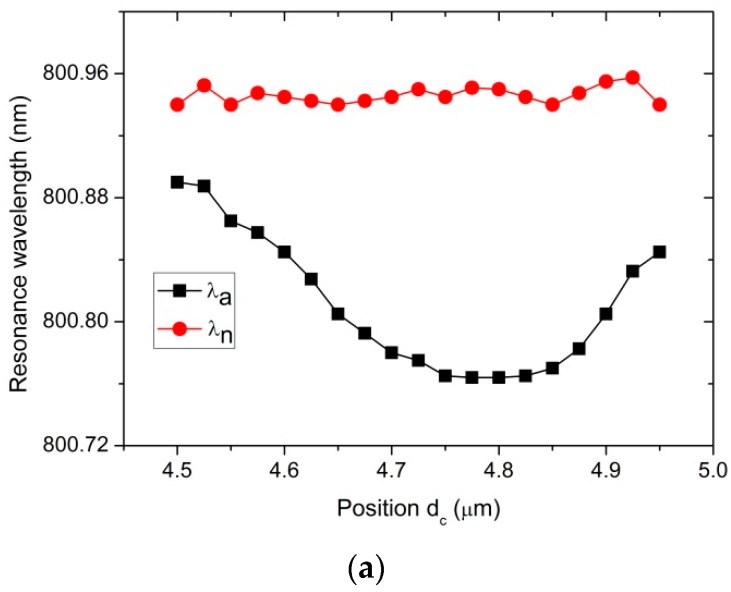

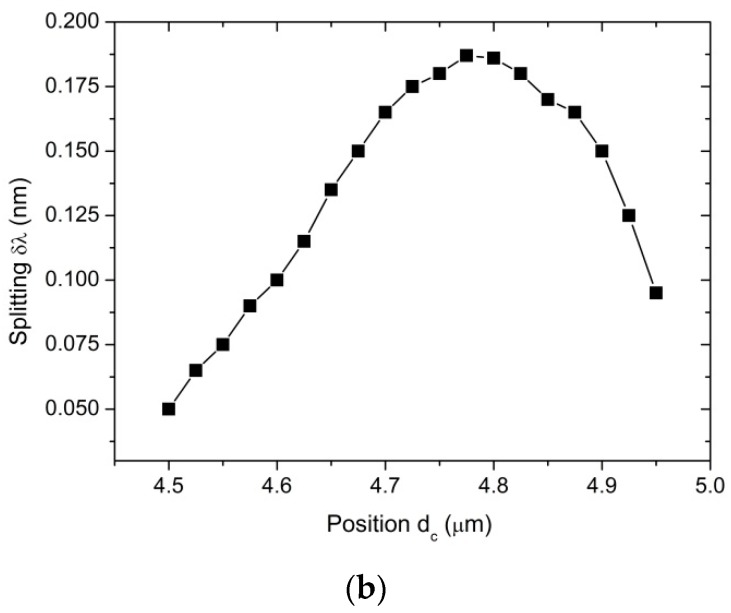
(Color online) Positional dependences of (**a**) resonance wavelength of the WGMs and (**b**) splitting of the resonance peaks ($\delta \lambda =\lambda_{n}-\lambda_{a})$. A nanocylinder of n_c_ = 1.5 and R_c_ = 50 nm is embedded. The reference wavelength of the unperturbed WGM is $\lambda_{o}=800.966\;\mathrm{nm}$.

This study examined the effects of the nanocylinder embedded inside the microcylinder (n_mc_ = 2.01) on the WGMs as a function of the position of the nanocylinder, as presented in Figure 1. The refractive index and radius of the nanocylinder are n_c_ = 1.5 and R_c_ = 50 nm, respectively. Figure 3a,b show the positional dependences of the resonance wavelength of the WGMs and the peak splitting, respectively.

As presented in Figure 3a, $\lambda_{a}$ markedly changes according to the position of the nanocylinder, whereas $\lambda_{n}$ was relatively unaffected. These results show that the shift in the resonance wavelength is associated with the magnitude of the electric field at the location of the nanocylinder. $\lambda_{n}$ shows a negligible dependence on $d_{c}$ because, for the WGM_n, the nanocylinder is moved along the line passing the node positions of the WGM. On the other hand, for the WGM_a, the nanocylinder passes the antinode positions of the WGM. In this case, the magnitude of the electric field that affects the resonance wavelength varies strongly with $d_{c}$. As can be clearly seen in Figure 3a, $\lambda_{a}$ is most strongly blue-shifted in the range $d_{c}=4.75\sim 4.85\;\mu m$ due to the large magnitude of the electric field in this region.

A noticeable decrease in $\lambda_{a}$ and a small decrease in $\lambda_{n}$ can be attributed to the smaller refractive index of the nanocylinder (n_c_ = 1.5) than the surrounding medium (n_mc_ = 2.01, microcylinder). The effective increase in the wavelength of the WGM_a inside the nanocylinder is compensated for by the decreased wavelength of the WGM_a in the microcylinder.

As plotted in Figure 3b, the splitting, $\delta \lambda =\lambda_{n}-\lambda_{a}$, shows a relatively broad peak centered at $d_{c}=4.775\;\mu m$. These results show that splitting is large in the region of strong electric fields. Based on these results, the location of the nanocylinder was chosen for further studies in the following sections.

### 3.2. Size Dependence

**Figure 4:**
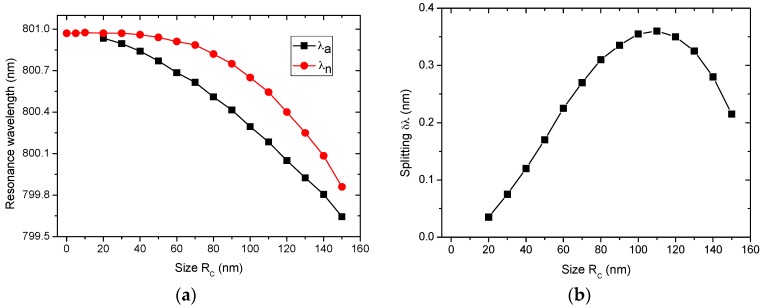
(Color online) Size dependence of (**a**) the resonance wavelength of the WGMs and (**b**) splitting of the resonance peaks ($\delta \lambda =\lambda_{n}-\lambda_{a}$). The nanocylinder of n_c_ = 1.5 is embedded at $d_{c}=4.85\;\mu m$. The reference wavelength of the unperturbed WGM is $\lambda_{o}=800.966\mathrm{nm}$.

In this study, a nanocylinder of n_c_ = 1.5 was embedded at $d_{c}=4.85\;\mu m$ and the size of the nanocylinder varied from 5 to 150 nm in radius. Figure 4a,b show the size dependence of the resonance wavelength and splitting, respectively.

Although both $\lambda_{n}$ and $\lambda_{a}$ are increasingly blue-shifted with increasing nanocylinder size, the splitting shows a broad peak, reaching a maximum of R_c_ = 110 nm. As shown in Figure 4a, when R_c_ < 10 nm, the perturbation due to the nanocylinder is too small to produce observable splitting.

Because the WGM_a is perturbed more strongly than the WGM_n for relatively small nanocylinders ($R_{c}=20\sim 110\;\text{nm}$), $\lambda_{a}$ decreases faster than $\lambda_{n}$ with increasing size. On the other hand, when $R_{c}$ > 110 nm, the nanocylinder cannot be treated as a small object compared to the wavelength of the WGM (~400 nm in the microcylinder). Interestingly, in this case, $\lambda_{n}$ decreases faster than $\lambda_{a}$, and the splitting becomes smaller. These results can be explained as follows.

**Figure 5:**
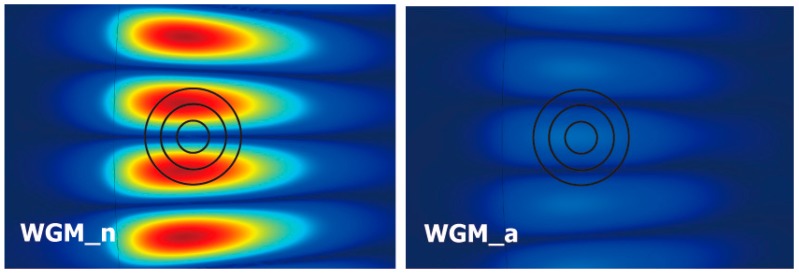
(Color online) Electric field distributions at the position of the nanocylinder for the two split WGMs. The circle indicate nanocylinders with $\left(R_{c}=50,\;75,\;\mathrm{and}100\;\text{nm}\right)$, respectively.

Figure 5 shows the intensity distributions of the electric field at the position of the nanocylinder for $R_{c}=50,\;100,$ and 150 nm. As displayed in Figure 5, the widths of the node and antinode regions are approximately 50 and 150 nm, respectively. When the nanocylinder is smaller than ~50 nm, it resides completely inside either the node region (WGM_n) or the antinode region (WGM_a). Therefore, for $\lambda_{n}<\sim 50\;\mathrm{nm}$, $\lambda_{n}$ is relatively constant while $\lambda_{a}$ decreases rapidly. As $R_{c}$ is increased to a size larger than 50 nm, the nanocylinder for the WGM_n gradually penetrates into the antinode regions and becomes increasingly perturbed. On the other hand, the nanocylinder for the WGM_a remains inside the antinode region and continues to be perturbed strongly until its size is large enough to begin penetrating into the node regions. On the other hand, once it begins to penetrate into the node regions, perturbation increases weakly with increasing $R_{c}$, and the decrease in $\lambda_{a}$ slows down. As a result, $\lambda_{n}$ approaches $\lambda_{a}$ for large nanocylinders. Furthermore, these combined effects give rise to a maximum of δλ at $R_{c}=110\;\text{nm}$, as shown in Figure 4b.

### 3.3. Refractive Index Dependence

**Figure 6:**
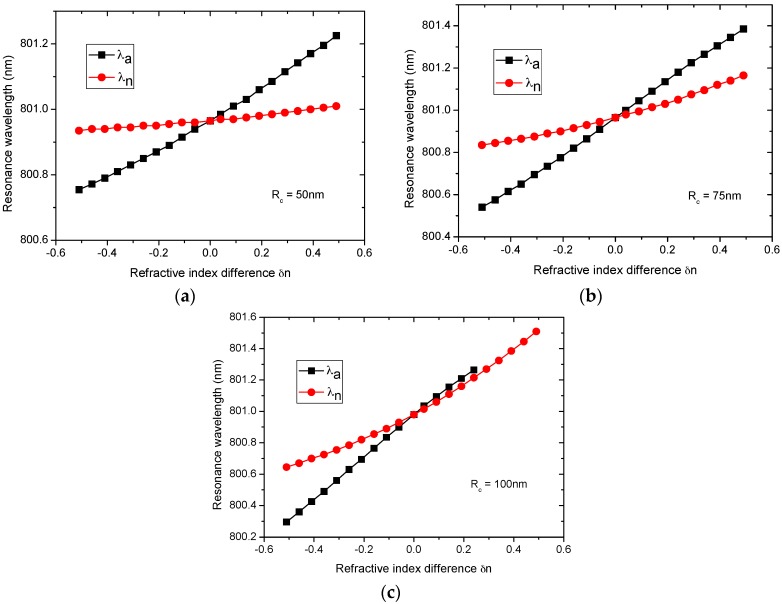
(Color online) Refractive index dependences of the resonance wavelength for (**a**) $R_{c}=50\;\text{nm}$; (**b**) $R_{c}=75\;\text{nm}$; and (**c**) $R_{c}=100\;\text{nm}$. The nanocylinder is embedded at $d_{c}=4.75\;\mu m$. The reference wavelength of the unperturbed WGM is $\lambda_{o}=800.966\;\mathrm{nm}$.

**Figure 7:**
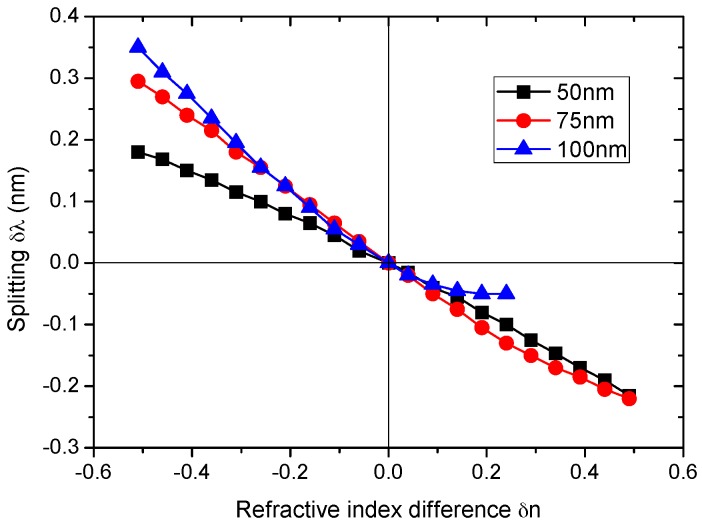
(Color online) Refractive index dependences of the splitting ($\delta \lambda =\lambda_{n}-\lambda_{a}$). The nanocylinder is embedded at $d_{c}=4.75\;\mu m$.

For this study, the nanocylinder was placed at $d_{c}=4.75\;\mu m$, and the refractive index of the nanocylinder was varied for three different sizes ($R_{c}=50,\;\;75,\;100\;\text{nm}$). The refractive index of the microcylinder was set to $n_{\text{mc}}=2.01$, and the refractive index difference is denoted as $\delta n=n_{c}-n_{mc}$. The resonance wavelengths and splitting for $R_{c}=50,\;75,\;100\;\text{nm}$ as a function of δn are illustrated in Figure 6a–c and Figure 7, respectively.

For all three cases, both $\lambda_{n}$ and $\lambda_{a}$ are blue-shifted for δn<0 and red-shifted for δn>0. As discussed previously, the shifts in the wavelength of the WGMs result from the refractive index contrast. For $R_{c}=50\;\text{nm}$, $\lambda_{n}$ varies almost linearly with $n_{c}$ for the entire range of $n_{c}$, whereas $\lambda_{a}$ shows weak nonlinear behavior with increasing |δn|. Furthermore, the slope of |δλ/δn| is larger for $\lambda_{a}$ than for $\lambda_{n}$. The shift in the wavelength of the WGMs has been investigated theoretically [1,7,8]. According to these works, the shift is proportional to the excess polarizability of the perturbing particle. Because the excess polarizability is not a linear function of the refractive index generally, the observed nonlinear behavior is expected.

The next part of the study examined how the size of the nanocylinder influences these behaviors. Both $\lambda_{n}$ and $\lambda_{a}$ for $R_{c}=75\;\text{nm}$ and 100 nm are observed to increase faster with increasing δn than for $R_{c}=50\;\text{nm}$ (larger |δλ/δn|). It can be inferred from these results that, for a given δn and $d_{c}$, a larger nanocylinder generally causes a larger shift in the resonance frequency. Interestingly, a plot of $\lambda_{n}$ deviates from linearity for $R_{c}=75\;\text{nm}$, and this nonlinear behavior is more apparent for $R_{c}=100\;\text{nm}$. Furthermore, for $R_{c}=100\;\text{nm}$, the curve of $\lambda_{a}$ as well as that of $\lambda_{n}$ shows nonlinear behavior in the range of δn>0. Because for δn>0.25, the linewidth of the WGM_n peak became larger than the splitting between the two peaks, $\lambda_{a}$ could not be determined in this range, as shown in Figure 6c.

In addition to the effects of the excess polarizability mentioned before, these nonlinear behaviors are also related to the degree of overlap of WGMs with the nanocylinder. As shown in Figure 5, the nanocylinder of $R_{c}=50\mathrm{nm}$ resides almost completely inside either the node or antinode region, resulting in a linear dependence of the resonance wavelengths on δn. However, as the size of the nanocylinder is increased, it penetrated gradually into the other regions and complicated nonlinear behavior appears, particularly for $\lambda_{n}$.

The splitting for the three cases shows several interesting features, as shown in Figure 7. First, the curve of the splitting for $R_{c}=50\;\text{nm}$ bends slightly downward over the entire range of δn. Second, in contrast, the curve of the splitting for $R_{c}=75\;\text{nm}$ shows almost linear behavior in the range, −0.5<δn<0.3, and deviates upward (0.3<δn<0.5). Third, for $R_{c}\;=100\;\text{nm}$, the curve shows almost linear behavior in the range of −0.5<δn<−0.1 and rapidly deviates from linearity for δn>−0.1.

In summary, the dependence of the splitting on δn is affected considerably by the nanocylinder size. In general, the nonlinearity of the curve increases with the increasing size of the nanocylinder. Furthermore, the curve tends to deviate from linearity more strongly for a positive δn than a negative δn. This point is important because pores and most impurity particles have the refractive index difference δn<0. The splitting can be used to develop sensors for determining the size of pores or impurity particles as well as the refractive index of unknown particles. For this purpose, the linear dependence of the splitting on either δn or the size is desirable. In general, these results show that our optical resonator system is suitable for detecting impurity particles that are smaller than the sizes of the node and antinode regions.

## 4. Conclusions

In this study, a two-dimensional (2D) microcylinder resonator $\left(Q\sim 2\times \;10^{5}\right)$ coupled to a waveguide was designed, and the finite element method was used to simulate the WGMs in the microcylinder resonator. This study focused on the effects of an embedded nanocylinder on the WGMs, particularly on the perturbation of the WGM resonances when varying the position, size, and refractive index of the nanocylinder.

Studying the positional dependence shows that the shift in the resonance wavelength is associated with the magnitude of the electric field at the location of the nanocylinder. Furthermore, the splitting displays a relatively broad peak centered at $d_{c}=4.775\;\mu m$, indicating that the splitting is large in the region of strong electric fields.

To study the size dependence, a nanocylinder of n_c_ = 1.5 was embedded at $d_{c}=4.85\;\mu m$, and the size of the nanocylinder was varied in the range of 5 to 150 nm in radius. Although both $\lambda_{n}$ and $\lambda_{a}$ are increasingly blue-shifted with increasing nanocylinder size, the splitting shows a broad peak at $R_{c}=110\;\text{nm}$. These results are attributed to the changing degree of overlap of the WGMs with the nanocylinder.

To study the dependence of the refractive index, the nanocylinder was placed at $d_{c}=4.75\;\mu m$, and the refractive index of the nanocylinder for three different sizes $\left(R_{c}=50,\;75,\;100\;\text{nm}\right)$ was varied. The refractive index dependences of splitting show linear behavior for a nanocylinder less than 50 nm in size, and the nonlinear behavior increases with the increasing size of the nanocylinder. These results generally show that our optical resonator system is suitable for detecting impurity particles that are smaller than the sizes of both node and antinode regions.

This system is not intended for detecting a single bacteria or viral particle. We intend to develop a sensor system with pores or nano-channels that many biological and chemical objects enter. For example, we can produce nano-channels (resembling the nanocylinder in our study) beneath the surface of a mycrocylinder. When biological and chemical objects move along nano-channels, they can be detected under strong WGM fields. Because the sizes of bacteria and viral particles are in the range 2–10 μm and 50–200 nm respectively, we can choose different diameters of microdisks and nano-channels depending on the size of detected particles.

This system can be compared with sensors based on surface plasmon resonance (SPR) [17,18,19]. The sensing mechanism of SPR is based on the measurement of the resonant peak shift caused by change in refractive index due to the binding of foreign species on the surface of metal thin films or nanoparticles. Because there are many different designs of biosensors, it is difficult to compare their sensitivities directly. The limit of detection (LOD) for the SPR biosensor is known to be in the range of ~pM to ~fM [20]. In comparison, LOD for the WGM biosensor having the same design with our system has been reported to be ~10 pM of DNA [21,22,23].

The pros of this system are a relatively high Q-value, efficient coupling, and extraction from the same optical fiber. On the other hand, the cons are the inherent complexity, the delicate alignment of the tapered fiber and the cavity, and the difficult microfabrication process of the cavity. However, these problems can be overcome by on-chip integrated systems in the near future. A technical solution to these problems was presented by Bog *et al.* [24,25,26].
